# 12α-Hydroxylated bile acid enhances accumulation of adiponectin and immunoglobulin A in the rat ileum

**DOI:** 10.1038/s41598-021-92302-z

**Published:** 2021-06-21

**Authors:** Reika Yoshitsugu, Hongxia Liu, Yoshie Kamo, Akari Takeuchi, Ga-Hyun Joe, Koji Tada, Keidai Kikuchi, Nobuyuki Fujii, Shinri Kitta, Shota Hori, Manami Takatsuki, Hitoshi Iwaya, Yasutake Tanaka, Hidehisa Shimizu, Satoshi Ishizuka

**Affiliations:** 1 Research Faculty of Agriculture, Hokkaido University, Kita-9, Nishi-9, Kita-ku, Sapporo, 060-8589 Japan; 2 Research Faculty of Fisheries, Hokkaido University, Hakodate, 041-8611 Japan; 3 Department of Bioscience and Biotechnology, Faculty of Agriculture, Kyushu University, Fukuoka, 819-0385 Japan; 4 Institute of Agricultural and Life Sciences, Academic Assembly, Shimane University, Matsue, 690-8504 Japan

**Keywords:** Sterols, Ileum, Antibodies

## Abstract

We previously reported that dietary supplementation with cholic acid (CA), the primary 12α-hydroxylated (12αOH) bile acid (BA), reduces plasma adiponectin concentration in rats. The aim of this study was to examine the distribution of adiponectin in the body of CA-fed rats and its influence on mucosal immunoglobulin A concentration in the intestine. Rats were fed a diet supplemented with or without CA (0.5 g CA/kg diet) for 13 weeks. A reduction in plasma adiponectin level was observed from week 3. At the end of the experiment, the CA diet reduced plasma adiponectin concentration both in the portal and aortic plasma. Accumulation of adiponectin was accompanied by an increase in cadherin-13 mRNA expression in the ileal mucosa of CA-fed rats. No increase was observed in adiponectin mRNA expression in the ileal and adipose tissues of the CA-fed rats. Immunoglobulin A concentration in the ileal mucosa was elevated in the CA-fed rats and was correlated with the ileal adiponectin concentration. 12αOH BAs may modulate mucosal immune response that are involved in the accumulation of adiponectin in the ileum.

## Introduction

Bile acids (BAs), produced from cholesterol in the liver, are amphipathic molecules that are involved in lipid absorption in the gut^1^. BAs are classified in several different ways, and the most well-known system divides them into primary and secondary BAs^1^. BAs that are newly produced in the liver are called primary BAs. BAs that are modified in their functional groups by gut microbes are referred to as secondary BAs. Other classifications of BAs are based on the placement and orientation of hydroxyl groups such as 12α-hydroxylation (12αOH). The major 12αOH BAs in rats include cholic acid (CA) and deoxycholic acid (DCA), whereas the non-12αOH BAs include chenodeoxycholic acid and muricholic acids^2^. A high-energy supply from diet enhances the production of 12αOH BA in the body. Such an increase in 12αOH BAs is frequently observed in mice^3^, rats^4,5^, and humans^6^.

In our previous study, we simulated a high 12αOH BA environment via CA-supplementation in the diet of rats and observed alterations in the gut microbiota^7^, hepatic steatosis without obesity, leaky gut, increased plasma transaminase activity, and reduced plasma adiponectin concentration^8^. Using multiple regression analysis, we also identified plasma adiponectin as a predictor of CA-induced hepatic steatosis^8^. Adiponectin is an adipokine that is mainly produced by adipose tissues and plays a role not only in energy metabolism but also in the prevention of atherosclerosis^9,10^. Accumulation of adiponectin has been reported in several organs and tissues, and is associated with the adiponectin receptor, cadherin-13/T-cadherin (CAD13)^10^.

Immunoglobulin A (IgA) serves as a barrier that protects the intestinal mucosa from external antigens^11^. Luminal antigens are recognized by immune cells such as dendritic cells (DCs) and macrophages in the Peyer’s patches (PPs)^11^ that are connected with mesenteric lymph nodes (MLNs) via the lymph ducts. DCs present antigens to T-cells in the MLN that triggers antibody production from plasma cells. IgA-producing plasma cells are selectively distributed in the mucosal area, including the intestine, and play a preventive role in mucosal infection^12^.

In this study, we evaluated the distribution of adiponectin in CA-fed rats and found an accumulation of adiponectin in the ileal mucosa in the CA-fed conditions. Additionally, we analyzed the association between adiponectin and IgA in the ileal mucosa.

## Results

### Alteration of fecal BAs and plasma parameters in rats fed CA diet (Study 1)

**Figure 1:**
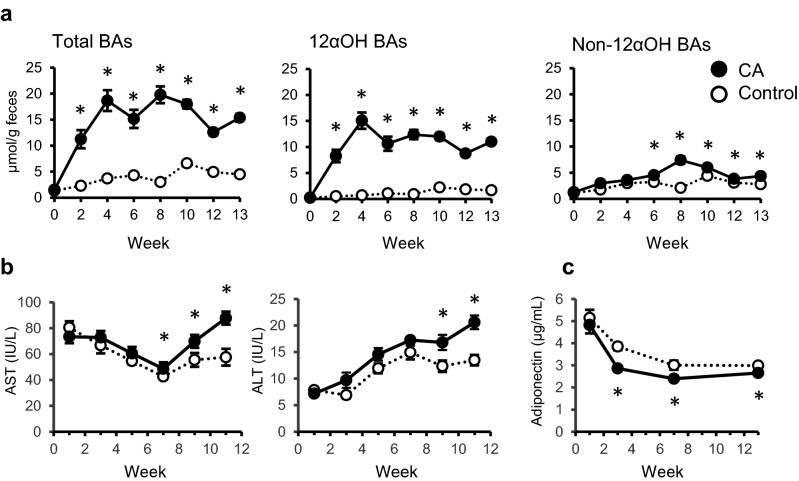
Changes in fecal BAs and plasma parameters in control and CA-fed rats (Study 1). (**a**) Fecal BA levels including total BAs (left), 12αOH BAs (middle), and non-12αOH BAs (right). (**b**) Changes in aspartate aminotransferase (AST) and alanine aminotransferase (ALT) activities. (**c**) Plasma adiponectin concentrations. Feces were collected throughout the day once every two weeks and at the end of the experimental period and total BAs, 12αOH BAs, non-12αOH BAs were measured as described in our previous study^8^. Plasma samples were isolated from blood collected from the tail veins. The values represent the means with standard errors of mean (SEM; n = 10). Open circles, control rats; filled circles, CA-fed rats. *P < 0.05 (compared to the control).

High levels of total BAs and 12αOH BAs were observed in the feces of CA-fed rats during the experimental period (Fig. 1a). An increase in non-12α BAs was observed in the CA-fed rats from week 6 onwards (Fig. 1a). An increase in aortic aspartate aminotransferase (AST) and alanine aminotransferase (ALT) levels was detected in the CA-fed rats from week 7 and week 9, respectively (Fig. 1b). There was a reduction in the systemic concentration of adiponectin in the blood from week 3 in the CA-fed rats (Fig. 1c). No difference was observed in fasting blood glucose levels between the two groups throughout the experimental period (Supplementary Table S1). There was no difference in food consumption, body weight, or relative organ weights of liver and visceral adipose tissues between the groups (Table 1). No difference was observed in inflammation-related genes such as *Tnfa*, *Cd163* (expressed in monocyte and macrophage marker), *Nos2* (nitric oxide synthase), *Nox2* (cytosolic subunit of neutrophil NADPH oxidase), and *p47* (a subunit of neutrophil NADPH oxidase) in ileal mucosa of the CA-fed rats (Supplementary Fig. S1a). There was a significant reduction in the expression of *p22* (a component of microbicidal oxidase system of phagocytes).

**Table 1:** Food intake, growth, and organ weights (Study 1).

	Control	CA
Cumulative food intake (g)	1546 ± 22	1505 ± 16
Final body weight (g)	374.2 ± 9.0	376.4 ± 5.5
**Organ weight (g/100 g body weight)**		
Liver	3.18 ± 0.06	3.20 ± 0.05
Visceral fat	6.55 ± 0.16	6.44 ± 0.18
Mesenteric fat	1.49 ± 0.04	1.61 ± 0.05
Epididymal fat	2.09 ± 0.06	1.93 ± 0.08
Retroperitoneal and perirenal fat	2.97 ± 0.10	2.89 ± 0.08

The rats were fasted for 16 h every two weeks from week 1 to 11 during the test period. Values are shown as mean ± SEM (n = 10).

### Distribution of adiponectin in relation to *Cad13* expression in rats fed CA diet (Study 2)

**Figure 2:**
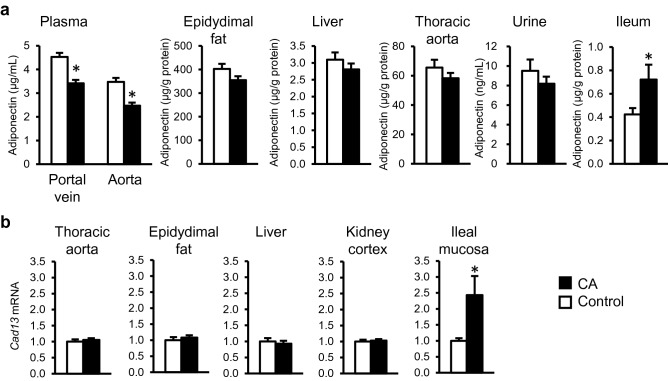
Distribution of adiponectin and cadherin-13 (*Cad13*) expression in the tissues in control and CA-fed rats (Study 2)*.* (**a**) Adiponectin concentration in tissues and fluid. (**b**) *Cad13* gene expression in tissues. The values represent the means with SEM (n = 12). Open bars, control rats; filled bars, CA-fed rats. *P < 0.05 (compared to the control).

Figure 2a shows the distribution of adiponectin in the body and urine of the rats. Adiponectin concentration was found to be reduced both in the portal and aortic plasma in the CA-fed rats at dissection and the concentration was higher in the portal plasma than in the aortic plasma in the control group (*P* < 0.05). No difference in adiponectin level was observed in epidydimal fat, liver, thoracic aorta, and urine, whereas a significant increase in adiponectin concentration was observed in ileal mucosa of CA-fed rats (*P* < 0.05). No difference was observed in adiponectin mRNA expression in epididymal fat, retroperitoneal fat, perirenal fat, mesenteric fat, thoracic aorta, and ileal mucosa (Supplementary Fig. S1b). There was a significant increase in *Cad13* mRNA expression in the ileal mucosa (*P* < 0.05), but not in the thoracic aorta, epidydimal fat, liver, and kidney cortex in the CA-fed rats (Fig. 2b).

**Figure 3:**
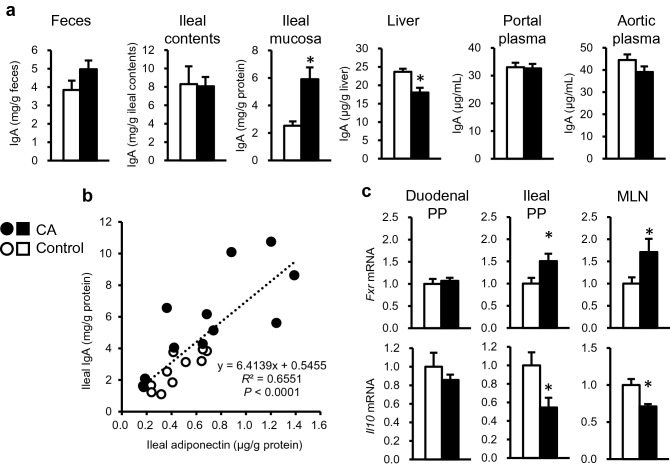
Distribution of immunoglobulin A (IgA), correlation with adiponectin level in the ileal tissues, and expression of receptors (Study 2). (**a**) IgA concentration in tissues and body fluids. (**b**) Pearson’s correlation between IgA and adiponectin concentrations in the ileal tissue. (**c**) Gene expression of farnesoid X receptor (*Fxr*) and interleukin-10 (*Il10*) in duodenal Peyer’s patch (PP), ileal PP, and mesenteric lymph nodes (MLN). The values represent the means with SEM (n = 12). Open circles, control rats; filled circles, CA-fed rats. *P < 0.05 (compared to the control).

Figure 3a shows the distribution of IgA in the body. A significant increase was observed in IgA concentration in the ileal mucosa (*P* < 0.05). In contrast, there was a reduction in hepatic IgA concentration (*P* < 0.05). There was no difference in the IgA concentration in feces, ileal contents, portal plasma, and aortic plasma. Further, we found a correlation between the concentrations of adiponectin and IgA in the ileal mucosa (*R*^2^ = 0.6551, *P* < 0.0001) (Fig. 3b). Gene expression analysis (Fig. 3c) revealed an increase in *Fxr* mRNA expression in the ileal PP and MLN in the CA-fed rats, whereas *Il10* mRNA expression was reduced at both sites (*P* < 0.05). No difference was detected in the expression of the genes in the duodenal PP. In tissue parameters in Study 2, there was significant increases in liver weight (12.0 ± 1.3 g in control, 14.4 ± 1.1 g in CA) and liver triglycerides (34.7 ± 5.2 mg/g liver in control, 86.7 ± 12.8 mg/g liver) in CA-fed rats. No difference was observed in final body weight and adipose tissue weights (data not shown).

## Discussion

We observed a reduction in plasma adiponectin concentration in CA-fed rats in our previous studies^7,13^, and confirmed the reduction not only in aortic plasma but also in portal plasma (Fig. 2a) in the present study. Although the adipose tissue is the major tissue that produces adiponectin^9^, we did not observe any difference in adiponectin mRNA expression in the adipose tissues of the CA-fed rats (Supplementary Fig. S1b). This observation suggests an alteration in the distribution of adiponectin protein in the body of the CA-fed rats. Actually, we observed selective accumulation of adiponectin in the ileal mucosa of the rats (Fig. 2a). Accumulation of adiponectin in a tissue is induced by the expression of CAD13, a specific receptor for the hexameric high-molecular weight forms of adiponectin, but not the other forms^14^, which plays a protective role in the injured tissues, as adiponectin exhibits anti-inflammatory effects via reduction of tumor necrosis factor-α (TNFα)^9,10^. One could suspect that the upregulation of *Cad13* and corresponding accumulation of adiponectin act to prevent the local BA toxicity and resolve the inflammatory responses in ileum. However, the ileal gene expression analysis in the inflammation-related genes (Study 1) suggest no sign of apparent inflammation or injury in this site (Supplementary Fig. S1b), suggesting that the ileal mucosal cells could tolerate the local BA overload. The accumulation of adiponectin (Fig. 2a) was accompanied by increased *Cad13* expression in the ileum of the CA-fed rats (Fig. 2b), which suggests upregulation of *Cad13* in response to the BA environment in the ileal mucosa. Presumably, CAD13 in the ileum binds to multimeric adiponectin thereby reducing the portal concentration of adiponectin partially and thus changing the adiponectin concentration in the ileum and portal plasma^15^.

Secondary BAs, such as DCA that alter cell membrane properties, are cytotoxic^16–18^. Our previous study showed high fecal excretion of DCA and its derivatives in CA-fed rats^8,19^. Prior to being excreted, some of these hydrophobic BAs diffuse passively in the colonic mucosa, trigger intracellular oxidative stress and other signal cascades, thus resulting in tissue injury. Given that the proportion of DCA was relatively low in 12αOH BAs in portal blood of CA-fed rats in our previous study^8^, other 12αOH BAs such as CA and taurocholic acid are possible mediator to induce plasma transaminase activities. Actually, the activities of both the plasma transaminases (AST and ALT) measured in this study were elevated from week 8 (Fig. 1a). On the other hand, we found a decrease in the plasma adiponectin concentration in CA-fed rats at week 3. This is much earlier than when elevated transaminase activities were observed in CA-fed rats. In BA metabolism in the CA-fed rats, we also observed an increase in fecal non-12αOH BAs from week 6. In our previous observation^8^, hepatic *Cyp7a1* expression is not suppressed in CA-fed rats regardless of CA supplementation. Those suggests that there was no apparent negative feedback inhibition in BA synthesis^20^. This CA-supplementation may mimic conditions such as increased 12αOH BA in obesity or a high-energy consumption in rats without inhibition of BA synthesis^4,5^.

Based on these results, we evaluated the role of adiponectin in the pathophysiology of CA-fed rats by analyzing IgA accumulation in the ileal mucosa to assess the influence of BA on mucosal immune responses in the ileum (Fig. 3a). Increased urinary excretion of adiponectin is plausible in the CA-fed rats because excretion of adiponectin has been reported previously in human IgA-nephropathy^21^. However, no increase in urinary adiponectin excretion was found in the CA-fed rats (Fig. 2a). Adiponectin is also a milk component^22^ and enhances cell proliferation in the MLN in rat neonates^23^. We observed enhanced gene expression of the BA receptor *Fxr* and decreased expression of *Il10*, a major immuno-regulatory cytokine in the MLN. In addition, the expression patterns in the ileal PP, but not the duodenal PP, were quite similar to those in MLN (Fig. 3c). Ileum is the major site of reabsorption of BAs in enterohepatic circulation^2^. In our previous observation^8^, there is a reduction in *Fxr* expression in ileal mucosa where no Peyer’s patch is included. In the present study, an increase was observed in *Fxr* expression in ileal Peyer’s patches. Those observations show different direction in modulation of *Fxr* expression depending on microenvironment in ileal tissue and suggest *Fxr* is not necessary in the IgA-producing cells in ileal mucosa where the accumulation of adiponectin was observed. BA environment may modulate functions of immune cells such as DCs or B-cells in the PPs. Local dysregulation of immune responses may be triggered by the uptake of abundant BAs. Such a BA-rich environment may influence the expression pattern of cytokines and chemokines in PP and MLN. Another possible factor that influences mucosal IgA concentration is leaky gut provoked by secondary BAs^24^. Our previous study also showed an increase in in vivo gut permeability in CA-fed rats with non-absorbable marker excretion in urine^8,13^. Leaky gut enhances the penetration of luminal antigens into the ileal mucosa that induces mucosal immune response^11^. Both BA-induced modulation of the immune response and leaky gut may concomitantly promote mucosal IgA concentration in the CA-fed rats**.**

In our previous study, the CA supplementation induces lipid accumulation in the liver accompanied by increase of liver weight^8^. On the other hand, fasting was repeated every 2 weeks in the present Study 1. Fasting induces autophagy in the liver^25^ via activation of peroxisome proliferator-activated receptor-α, which results in lipolysis. Repeated induction of autophagy by fasting once every 2 weeks may lead to energy expenditure in the liver of CA-fed rats in the present study. Responses that observed in the CA-fed rats may reflect a border between physiology and pathology in general.

In conclusion, CA-supplementation in the diet reduced plasma adiponectin concentration in rats, whereas it caused adiponectin accumulation in the ileal mucosa that correlated with IgA concentration in the ileal mucosa. Abundant 12αOH BAs in enterohepatic circulation may be a modulator of IgA-producing cell functions in the gastrointestinal tract.

## Materials and methods

### Animals

The present study was approved by the Institutional Animal Care and Use Committee of National Corporation, Hokkaido University (approval number: 14–0026 and 17–0119), and all animals were maintained in accordance with the Hokkaido University Manual for Implementing Animal Experimentation. The study was carried out in compliance with the ARRIVE guidelines. WKAH/HkmSlc male rats (3-week-old) were purchased from Japan SLC (Shizuoka, Japan). They were housed in an air-conditioned room at 22 ± 2 °C with 55 ± 5% humidity and light period from 08:00 to 20:00 h. The rats were housed individually in wire-bottomed cages and allowed ad libitum access to diet and water. Body weight and food consumption were measured every two days. The rats were acclimatized to a control diet based on the AIN-93G formulation (Table 2)^26^.

**Table 2:** Diet compositions.

	Control	CA
	g/kg diet	
Dextrin^a^	529.5	529.5
Casein^b^	200.0	200.0
Sucrose^c^	100.0	99.5
Soybean oil^d^	70.0	70.0
Cellulose^e^	50.0	50.0
Mineral mixture^f^	35.0	35.0
Vitamin mixture^g^	10.0	10.0
Choline bitartrate^h^	2.5	2.5
L-Cystine^h^	3.0	3.0
Cholic acid	–	0.5

^a^TK-16 (Matsutani Chemical Industry Co., Ltd., Hyogo, Japan).

^b^NZMP Acid Casein (Fonterra Co-Operative Group Limited, Auckland, New Zealand).

^c^Nippon Beet Sugar Manufacturing Co.,Ltd., Tokyo, Japan.

^d^J-Oil Mills, Inc., Tokyo, Japan.

^e^Microcrystalline cellulose (Ceolus PH-102, Asahi Kasei Corporation, Tokyo, Japan).

^f^AIN-93G mineral mixture (MP Biomedicals, USA).

^g^AIN-93 vitamin mixture (CLEA Japan, Inc., Tokyo, Japan).

^h^Wako Pure Chemical Industries, Ltd. Osaka, Japan.

### Alteration in fecal BAs and plasma parameters in rats fed CA diet (Study 1)

The acclimatized rats were then divided into two groups and fed either control diet (n = 10) or a diet supplemented with CA at 0.5 g/kg diet (n = 10) for 13 weeks. Tail vein blood was collected from the rats every two weeks from week 1 to 11 during the test period from 9:00 to 12:00 after food deprivation for 16 h, to measure fasting plasma glucose concentration. In addition, feces were collected for 24 h every two weeks from week 2 to 12 and in week 13 to measure fecal BA composition. At the end of the experimental period, blood was collected from the aorta abdominalis under anesthesia with sodium pentobarbital (Somnopentyl, 50 mg/kg body weight, Kyoritsu Seiyaku Corporation, Tokyo, Japan). An anticoagulant reagent (heparin, 50 U/mL blood, Nakarai Tesque Inc., Kyoto, Japan) and a protease inhibitor (aprotinin, 500 KIU/mL blood, Fujifilm Wako Pure Chemical Corporation, Osaka, Japan) were added to the collected blood and the plasma was separated. The rats were killed by exsanguination thereafter. The liver, visceral fat, and intestinal contents were collected and weighed. The ileal mucosa was collected for mRNA expression analysis of inflammation-related genes.

### Distribution of adiponectin in relation to cadherin-13 expression in rats fed CA diet (Study 2)

A 13-week feeding experiment was conducted using WKAH/HkmSlc rats under the same experimental conditions (n = 12 each for control and CA), except that the rats were maintained without food deprivation. At the end of the experimental period, blood was collected from 9:00 to 12:00 from the portal vein and aorta abdominalis under anesthesia with Somnopentyl. As mentioned before, heparin and aprotinin were added to the collected blood and the plasma was separated. The rats were killed by exsanguination thereafter, and urine in the urinary bladder was collected. The ileal contents, MLN, PP (ileum and duodenum), ileal mucosa, liver, kidney, epididymal fat, and thoracic aorta were collected. Feces were collected for 24 h at the end of the experimental period. Messenger RNA expression was analyzed of adiponectin (epidydimal fat, perirenal fat, retroperitoneal fat, mesenteric fat, thoracic aorta, and ileal mucosa) and CAD13 (thoracic aorta, epidydimal fat, liver kidney cortex, and ileal mucosa).

### BA analysis

The amount of each BA in the feces was measured after extraction as previously reported^27^. Total BAs, 12αOH BAs, and non-12αOH BAs were measured^8,28^ with nordeoxycholic acid (23-nor-5β-cholanic acid-3α,12α-diol) as an internal standard. The molecular species in 12αOH BAs, and non-12αOH BAs were shown in our previous report^29^.

### Measurement of biochemical parameters

Adiponectin, IgA, and glucose were analyzed using Mouse/Rat Adiponectin ELISA Kit (Otsuka Pharmaceutical Co. Ltd., Tokyo, Japan), Rat IgA ELISA kit (Bethyl Laboratories Inc., Montgomery, TX, USA), and Glucose CII-test Wako (Wako Pure Chemical Industries Ltd., Osaka, Japan), respectively. Transaminase CII-test Wako (Wako Pure Chemical Industries) was used to determine the aspartate aminotransferase (AST)/alanine aminotransferase (ALT) activities.

### Gene expression analysis

Gene expression analysis was performed as previously described^30^. Tissues used for mRNA isolation were stored at − 80 °C until use. Total RNA was isolated from the tissues using an RNeasy Mini Kit (Qiagen, Hilden, Germany). The RNeasy Plus Universal Midi Kit (Qiagen) was used, especially for fat tissues, according to the manufacturer’s instructions. Complementary DNA was synthesized from 1 µg of RNA using ReverTraAce qPCR RT master mix with gDNA remover (Toyobo Co. Ltd., Osaka, Japan) according to the manufacturer’s instructions. Quantitative PCR was performed using an Mx3000P real-time PCR system (Agilent Technologies, Santa Clara, CA, USA) with TaqMan Gene Expression Assays (Thermo Fisher Scientific, Waltham, MA, USA) as follows: Rn03302271_gH for ribosomal protein lateral stalk subunit P0 (*Rplp0*), Rn00562055_m1 for tumor necrosis factor-α (*Tnfa*), Rn01492519_m1 for CD163, Rn00561646_m1 for nitric oxide synthase 2 (*Nos2*), Rn00576710_m1 for NADPH oxidase 2 (*Nox2*), Rn00586945_m1 for p47, Rn00577357_m1 for p22, Rn00572658_m1 for farnesoid X receptor (*Fxr*), and Rn00563409_m1 for interleukin-10 (*Il10*). We performed quantitative real-time PCR for *Cad13* expression analysis using Syber Premix Ex Taq II Green (Takara Bio Inc., Shiga, Japan) according to the manufacturer’s protocol using the following oligonucleotide primers: rat *Rplp0*, 5′-GGCAAGAACACCATGATGCG-3′ (forward) and 5′-GTGATGCCCAAAGCTTGGAA-3′ (reverse); rat *Cad13*, 5′-AGTGAATGCCACAGACCCG-3′ (forward) and 5′-AATCCGTCACCATGATGGGC-3′ (reverse). Relative expression levels for each sample were calculated after normalization to those of RPLP0 mRNA as a reference gene using the standard curve method.

### Statistical analyses

The significance of differences between control and CA groups was determined using Student’s *t*-test. Pearson’s correlation was used to analyze the relationship between ileal concentrations of IgA and adiponectin. A *P* value < 0.05 was considered to be significant. JMP version 14.0.0 (SAS Institute Inc., Cary, NC, USA) was used for the statistical analyses.

## Supplementary Information

Supplementary Information.

## Abbreviations

12αOH: 12α-Hydroxylated
ALT: Alanine aminotransferase
AST: Aspartate aminotransferase
BA: Bile acid
CA: Cholic acid
CAD13: Cadherin-13
DC: Dendritic cell
DCA: Deoxycholic acid
FXR: Farnesoid X receptor
IgA: Immunoglobulin A
IL10: Interleukin-10
MLN: Mesenteric lymph node
NOS2: Nitric oxide synthase 2
NOX2: NADPH oxidase 2
PP: Peyer’s patch
RPLP0: Ribosomal protein lateral stalk subunit P0
TNFα: Tumor necrosis factor-α
