# Supplementary material for: 12α-Hydroxylated bile acid enhances accumulation of adiponectin and immunoglobulin A in the rat ileum
Source: Sci Rep. 2021 Jun 21;11:12939. doi: 10.1038/s41598-021-92302-z (PMC8217220; doi:10.1038/s41598-021-92302-z)
Supplement: Supplementary file 1 — Supplementary Information. [file 41598_2021_92302_MOESM1_ESM.pdf]

12α-hydroxylated bile acid enhances accumulation of adiponectin and immunoglobulin A in the rat ileum

Reika Yoshitsugu [1], Hongxia Liu [1], Yoshie Kamo [1], Akari Takeuchi [1], Ga-Hyun Joe [1,2], Koji Tada [1], Keidai Kikuchi [1], Nobuyuki Fujii [1], Shinri Kitta [1], Shota Hori [1], Manami Takatsuki [1], Hitoshi Iwaya [1], Yasutake Tanaka [1,3], Hidehisa Shimizu [1,4], and Satoshi Ishizuka [1,*]

[1] Research Faculty of Agriculture, Hokkaido University, Sapporo 060-8589, Japan

[2] Research Faculty of Fisheries, Hokkaido University, Hakodate 041-8611, Japan

[3] Department of Bioscience and Biotechnology, Faculty of Agriculture, Kyushu University, Fukuoka 819-0385, Japan

[4] Institute of Agricultural and Life Sciences, Academic Assembly, Shimane University, Matsue 690-8504, Japan

[*] Corresponding author: Research Faculty of Agriculture, Hokkaido University, Kita-9, Nishi-9, Kita-ku, Sapporo 060-8589, Japan

Email: zuka@chem.agr.hokudai.ac.jp

Supplementary Table S1. Fasting blood glucose levels (Study 1)

Week	Control	CA
	mg/dL	
1	103 ± 4	99 ± 5
3	102 ± 1	108 ± 3
5	103 ± 4	102 ± 5
7	98 ± 5	94 ± 3
9	91 ± 2	91 ± 2
11	86 ± 2	85 ± 2

Values are shown as means ± SEM (n=10). Tail vein blood was withdrawn after 16 h of food deprivation.

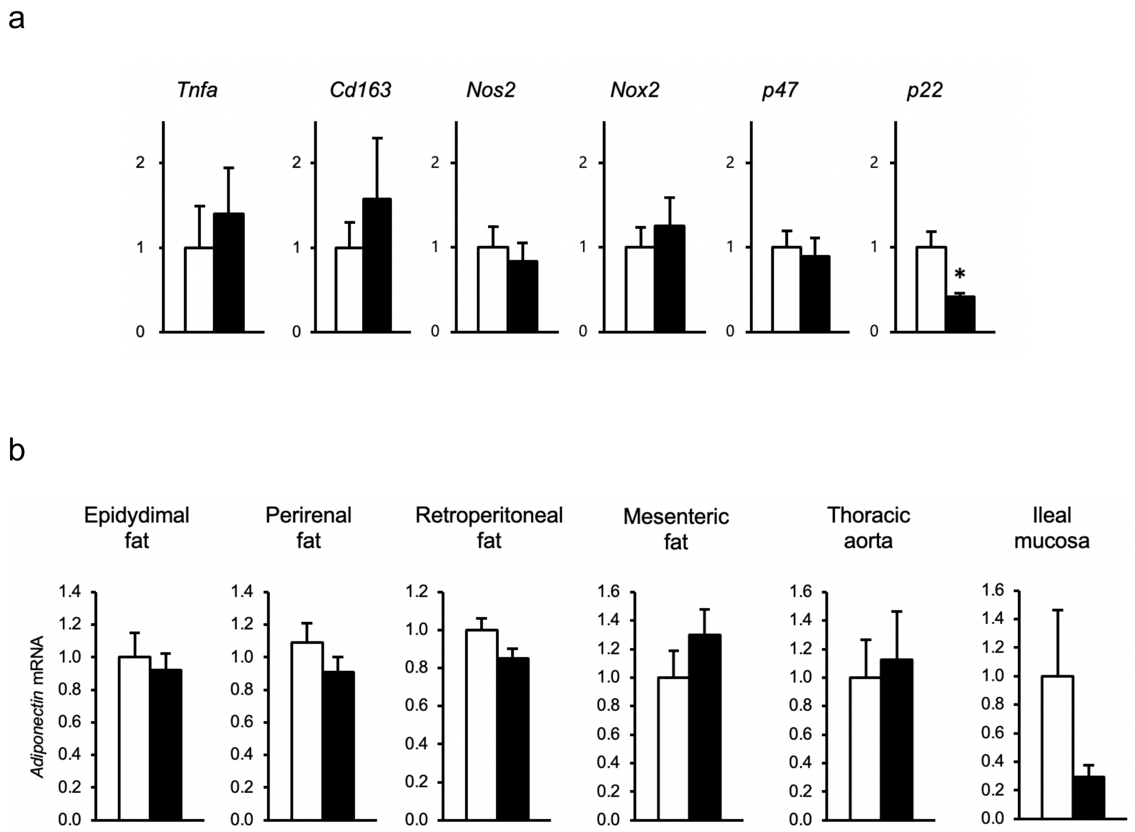

Supplementary Figure S1. Expression of mRNAs in ileal mucosa and adiponectin mRNA in tissues in control and CA-fed rats. (a) Messenger RNAs of inflammation-related genes in ileal mucosa of rats (n = 10) (Study 1). (b) Adiponectin mRNA in several tissues (n = 12) (Study 2). The values represent the means with SEM. Open bars, control rats; filled bars, CA-fed rats. * $P <$ 0.05 (compared to the control).
